# Virus Diseases of Economic Importance on Food Legumes in Africa and Their Control

**DOI:** 10.3390/v17121555

**Published:** 2025-11-28

**Authors:** Adane Abraham

**Affiliations:** Department of Biological Sciences and Biotechnology, School of Life Sciences, Botswana International University of Science and Technology, Private Bag 16, Palapye 100171, Botswana; abrahama@biust.ac.bw

**Keywords:** symptoms, vectors, geographical distribution, host range, yield losses, virus properties

## Abstract

Virus diseases are among the major constraints in the production of food legumes in Africa, causing substantial crop losses. Common bean mosaic and black root, cowpea mosaic, chickpea stunt, faba bean necrotic yellows and stunt, groundnut rosette, and soybean mosaic are the six diseases considered economically significant in Africa. Past research enabled the description of the main characteristics of the causal viruses, including particle and genome properties, modes of transmission, host range, and virus–vector relationships. Such information in many cases assisted in developing effective diagnostics and disease management methods such as host resistance, chemical vector control, and cultural practices. Integrating two or more of these approaches is usually more effective. The major challenge, however, remains ensuring the adoption of such recommendations at a sufficiently large scale by many farmers to have an impact over wider geographical areas. Future work should focus on scaling up the adoption of available control technologies and generating new information, including epidemiological data, to support future management decisions. Furthermore, since the occurrence and significance of viruses on food legumes in many African countries are still not studied, large-scale surveys to identify viruses, establish their distribution and impact, and working out suitable control measures are required.

## 1. Introduction

Food legumes (Fabaceae) are important components of farming systems worldwide, acting as rich sources of protein and edible oil. A variety of indigenous and introduced legume crops are cultivated all over Africa, where they play a critical role in food and nutritional security, income generation, and soil fertility, particularly for smallholder farmers. The major legumes grown in Africa are cowpea (*Vigna unguiculata* (L.) Walp.), chickpea (*Cicer arietinum* L.), groundnut (*Arachis hypogaea* L.), common bean (*Phaseolus vulgaris* L.), soybean (*Glycine max* (L.) Merr, and faba bean (*Vicia faba* L.) [1]. The crops are cultivated for consumption as grains and, to a lesser extent, as green vegetables. Cowpea, groundnut, common bean, and soybean are primarily cultivated as warm-season food legumes mostly in sub-Saharan Africa (SSA), whereas chickpea and faba bean are cool-season food legumes grown predominantly in countries of Northeast and Northern Africa. These six food legumes are cultivated in Africa annually in a total area of over 49.5 million hectares, with over 44.7 million tons of production as detailed in Table 1 [1]. The production of food legumes is challenged by several abiotic and biotic constraints such as pests and diseases, including those caused by viruses. Legumes are naturally infected by at least 168 viruses globally [2], many of which cause serious diseases that reduce crop yield and quality [3,4]. A comprehensive review of research on virus diseases of economic significance on food legumes grown in Africa is not available, as previous publications are limited in scope to either individual crops, groups of crops, or specific regions of the continent. In this paper, available information on economically important virus diseases of six major food legume crops and their available control options is reviewed. The key characteristics of the viruses involved in these diseases, such as particle morphology, genome type and size, and geographical distribution within the continent, are presented in Table 2.

## 2. Bean Common Mosaic and Black Root Disease

Common bean is widely grown in many African countries, where it is an important part of the diet as a rich source of protein. About 28% and 29% of global common bean cultivation areas and production as grain, respectively, comes from Africa [1]. Countries of SSA, namely Tanzania, Kenya, Uganda, Rwanda, Burundi, Cameroon, Ethiopia, the Democratic Republic of Congo, Malawi, and Benin, are the top ten producers [1]. The most important viruses of common bean in Africa are bean common mosaic virus (BCMV) and bean common mosaic necrosis virus (BCMNV) (both genus *Potyvirus*, family *Potyviridae*) [5]. Formerly, virus isolates belonging to BCMNV and BCMV used to represent strains in serotypes A and B of BCMV, respectively, but due to their genetic and biological difference, they were subsequently reclassified as distinct viral species named BCMNV and BCMV [24]. BCMV now includes several strains including some previously considered distinct virus species such as blackeye cowpea mosaic virus from cowpea (BCMV-BlCM strain) and peanut stripe virus (BCMV-PSt strain) in groundnut [5].

**Figure 1:**
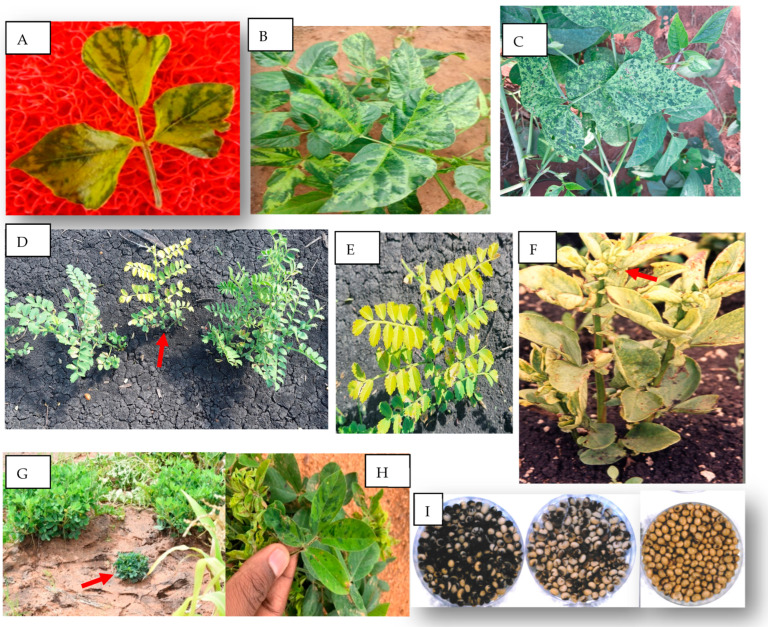
Symptoms of virus diseases on various legumes in Africa. (**A**) Mosaic, interveinal chlorosis, and distortion on leaves of wild bean (*Macroptilium atropurpureum*) caused by bean common mosaic virus (Palapye, Botswana). Similar symptoms are caused in common beans by the virus. (**B**,**C**) Variants of mosaic and mottle symptoms on cowpea caused by cowpea aphid-borne mosaic virus (Palapye, Botswana). (**D**) Stunting and yellowing on young kabuli chickpea plant (in the middle, red arrow) caused by a polerovirus compared to two healthy plants in the left and right (Pandamatenga, Botswana). (**E**) Close photo of the stunted plant in (**D**) showing leaf yellowing. (**F**) Faba bean plant with yellowing, leathery leaf, and stunting with initial necrosis (red arrow) caused by faba bean necrotic stunt virus (Ambo, Ethiopia). (**G**) Severe stunting and rosette symptoms (red arrow) caused by groundnut rosette disease complex, note the two nearby healthy groundnut plants with normal growth (Nata, Botswana). (**H**) Close photo of mosaic, mottle, and leaf distortion symptom caused by groundnut rosette disease complex (Palapye, Botswana). (**I**) Mottling of soybean seeds harvested from plants infected by soybean mosaic virus; left: blackish mottling in cultivar Williams, middle: brown mottling in cultivar Clark, and right: healthy unmottled seeds.

BCMV and BCMNV cause similar disease symptoms in susceptible common bean cultivars, which include mosaic, leaf curling, mild yellowing, and malformation with general stunting. In addition, BCMNV causes the lethal “black root” disease in varieties possessing the dominant I resistance gene [25]. This disease is characterized by red-brown expanding spots on leaves that develop into systemic necrosis, which often leads to the death of the plants upon early infection. Yield losses due to BCMV and BCMNV infection are significant, reaching up to 80% [25], while yield losses of up to 50% were reported in Morocco [26]. Although both viruses are major constraints on bean production and can cause serious crop losses in Africa, BCMNV is economically more important than BCMV in much of Central, Eastern, and Southern Africa [5,6]. Both BCMV and BCMNV are transmitted by aphids in a non-persistent manner, the most important vector species in many countries being *Acyrthosiphon pisum*, *Aphis fabae*, and *Myzus persicae* [27]. The viruses are also seed-transmitted, infected seeds often being responsible for primary infections in the crop. Disease outbreaks can be triggered either by using contaminated seed, which provides initial inoculum that can be further spread by aphids, or the viruses may infect a healthy crop population when viruliferous aphids immigrate from infected wild plants [5,28]. BCMV and BCMNV are commonly found in wild legumes (Figure 1A) and weeds around and within smallholder farmers’ fields in Africa, and BCMNV is thought to have evolved from BCMV there [6,28].

The most effective method to control BCMV and BCMNV is the use of resistant varieties [25]. Sources of resistance are available for breeding varieties of common bean resistant to BCMV and BCMNV. The deployment of the dominant I gene, coupled with the recessive resistance gene bc-3, provides effective resistance in bean cultivars to all known isolates of BCMV and BCMNV in Africa, particularly in Central and East Africa where BCMNV is endemic [25,29]. Bean cultivars with the dominant I gene are resistant to BCMV but susceptible to BCMNV because the virus triggers systemic necrosis (black root disease) in plants possessing the gene. When resistant varieties are not available, certified virus-free seeds can be an effective means of control, as practiced in the bean seed program in California, where a limit of 0.5% BCMV has aided in the control of the disease [30]. Although the use of virus-free bean seeds is probably a cost-effective management measure, it is not practiced by farmers in Africa due to the lack of clean seed production schemes [5]. Future research should explore the tolerance level of seed-borne inoculum, isolated areas for production of virus-free seeds, and the use of clean seeds in integration with other methods such as resistance, cultural practices, and vector control.

## 3. Cowpea Mosaic Disease

Cowpea is the most widely cultivated indigenous food legume in Africa, grown mainly by subsistence farmers. It is grown throughout SSA, which accounts for about 98.3% and 96.8% of global cultivation area and production, respectively, [1]. Most of this production is confined to West Africa with Nigeria, Niger, and Burkina Faso alone contributing 82% and 83% of global cultivation area and production, respectively, although many other countries in western, central, eastern, and southern Africa are also significant growers [1]. Virus diseases of cowpea are recorded wherever the crop is cultivated. At least 18 viruses infect cowpea in Africa [31], of which those causing mosaic type of symptoms such as vein banding, dark green and yellow mosaic, interveinal chlorosis, mottling, leaf distortion, and stunting are the most common (Figure 1B,C) [32]. Virus diseases cause yield losses of 20–80% in Africa [7,32]. Symptom type, severity, and associated yield losses are influenced by cowpea genotype, virus and strain, and time of infection. The most widespread and economically important viruses associated with mosaic diseases are cowpea aphid-borne mosaic virus (CABMV), bean common mosaic virus-black eye cowpea strain (BCMV-BlCM); formerly black eye cowpea mosaic virus (both in genus *Potyvirus,* family *Potyviridae*), and cucumber mosaic virus (CMV, genus *Cucumovirus,* family *Bromoviridae*) [7,8]. Infection of CABMV is reported to have caused complete losses of the cowpea crop in northern Nigeria [9]. CABMV and BCMV produce indistinguishable mosaic type symptoms on susceptible cowpeas, while CMV causes mild mosaic or symptomless infection depending on the cowpea cultivar. Mixed infections of two or more of these viruses cause more serious damage, including premature death due to synergistic effects, as reported for CABMV and CMV in Morocco [33] and Nigeria [34]. All three viruses are transmitted non-persistently by different aphids such as *Aphis craccivora* and *Myzus persicae,* and seed-transmitted to varying extents [27]. Two other mosaic-causing viruses of economic importance in some locations in Africa are the whitefly-transmitted cowpea mild mottle virus (CPMMV) in southwestern Nigeria [35] and the beetle-transmitted southern bean mosaic virus (SBMV) [36]. Both these viruses are also seed-transmitted.

The best approach to control cowpea mosaic diseases is the use of host resistance. Resistance in cowpea to single infections of CABMV or BCMV-BlCM was found in germplasm accessions TVu401, TVu1453, and TVu1948, and in breeding lines IT82D-885, IT28D-889, and IT82E-60 [37]. However, since mixed virus infections are common in cowpea fields and often cause more severe symptoms due to synergistic interactions, efforts have been made to obtain multiple virus resistance to different virus species [3,7]. Multiple resistance to BCMV-BlCM, CMV, and SBMV has been identified in breeding lines IT98K-1092-1, and IT97K-1042-3 [8,38]. In addition, since all mosaic-inducing viruses are seed-borne in cowpea, the use of virus-free seeds is recommended as an important step in disease control [3]. However, practical steps to provide farmers with certified virus-free cowpea seeds in Africa are lacking. Future research should prioritize the establishment of virus-free seed production and supply strategies so that the introduction of seed-borne inoculum to the field and further spread by insect vectors is prevented. Chemical control of insect vectors by using pesticides and cultural measures are believed to contribute to management efforts, but they are seldom feasible for smallholder farmers in SSA. The occurrence, relative importance of cowpea viruses, and their impacts are not yet well documented in many countries (e.g., in southern Africa), and future research should address this gap.

## 4. Chickpea Stunt Disease

Chickpea is widely grown in eastern and northern parts of Africa including Ethiopia, Sudan, Algeria, Morocco, Tunisia, Tanzania, and Uganda [1]. Ethiopia contributes to more than half of the total chickpea production in Africa. The most economically important viral disease of chickpea in the region is stunt, which can cause up to 95% yield losses [39]. In eastern and northern Africa, chickpea stunt is mostly caused by two poleroviruses, chickpea chlorotic stunt virus (CpCSV) and beet western yellows virus (BWYV), both in the genus *Polerovirus*, family *Solemoviridae*, singly or sometimes in mixed infection [10,11,40]. Symptoms caused by CpCSV and BWYV on chickpea are indistinguishable and include stunting, shortening of internodes, reduction in leaf lamina, bushy and brittle appearance of plants, and leaf yellowing in kabuli or reddening in desi types with poor or no pod setting (Figure 1D,E). Other phloem-limited viruses such as chickpea chlorotic dwarf virus (CCDV), faba bean necrotic yellows virus, and bean leafroll virus also naturally infect chickpea in the region, causing stunt, but usually occur at very low incidence [11,40,41]. CCDV is the major cause of chickpea stunt resulting in huge crop losses in South Asia, including India [42].

CpCSV was first reported from Ethiopia on chickpea, faba bean, and other legumes and has subsequently been reported across North Africa, the Middle East, and parts of Central Asia [10,43,44]. Epidemics of stunt caused by CpCSV on chickpea have been reported from Ethiopia, Tunisia, and Syria [39,40]. The virus is transmitted persistently by the aphid species *Aphis craccivora* and *Acyrthosiphon pisum* but not by seed [43,44]. CpCSV has been reported in nearly all chickpea-growing countries in Northeast and North Africa, and West and Central Asia, where surveys were conducted, but not from other parts of the world including the Indian subcontinent, Europe, the Americas, Australia, or the Far East [39]. BWYV, on the other hand, has a wider global distribution and is reported by most countries growing chickpea [12,13]. It has a very wide natural host range in more than 20 families, including many in Fabaceae, Brassicaceae, and Compositae, and infects chickpea and several other legume and non-legume plants worldwide [11,27]. It can cause yield losses on chickpea ranging from 8 to 90% [45]. BWYV is transmitted by aphids in a persistent, non-propagative manner, the main vectors being *M. persicae*, *A. craccivora*, *A. pisum,* and *Aulacorthum solani* [27]. Whereas BWYV is among the most common viruses detected in chickpea and other legumes in Africa, recent sequence-based studies indicate that many polerovirus isolates from chickpea, lentil, and faba bean from Africa and Australia previously considered as BWYV isolates by serological tests belong to *Turnip yellows virus* or other polerovirus species [46,47,48,49]. This is believed to be due to serological cross-reaction of BWYV antibodies used in those studies with several other poleroviruses, which hampered accurate virus identification in the samples. Hence, more thorough investigations including genome sequencing and biological properties of legume BWYV-like isolates are needed in Africa and other parts of the world to determine the exact species infecting food legumes to guide effective diagnosis and resistance breeding.

No resistant chickpea variety is currently available commercially for CpCSV and BWYV control in the region, but sources of resistance have recently been identified. For instance, a few genotypes of chickpea (e.g., IG 69719) tolerant to the local CpCSV isolate have been reported in Tunisia [50], whereas more recently, six chickpea genotypes (IG9000, IG69434, IG69656, IG69693, IG71832, and IG128651) identified as resistant or tolerant to CpCSV in Syria were recommended for use as resistance sources [51]. It remains to be seen whether the utilization of such resistance sources could lead to CpCSV control in the field since virus strains could differ. Seed treatment with two pesticides, Celest Top and Apron Star 45WS, significantly reduced field spread and yield loss due to BWYV and CpCSV compared with untreated seeds in Tunisia [50]. Such pesticides can be incorporated as a component in developing integrated virus management options to reduce losses. Until CpCSV- and BWYV-specific control options are made available by research; however, a combination of control measures used for the management of better-studied phloem-limited viruses with similar modes of spread (e.g., persistent transmission by aphids) can be used [4,40]. After fine-tuning as needed, such interim approach employing available generic information from other related systems that has been successfully used as an integrated virus disease management strategy for grain legumes in countries like Australia may be adopted in Africa.

## 5. Faba Bean Necrotic Yellows and Stunt Disease

Faba bean is a major food legume crop in northeastern and northern Africa, with Ethiopia, Egypt, Sudan, Tunisia, Algeria, and Morocco being the major producers. Africa contributes 27% and 26% of global cultivated areas (hectarage) and production (tons), respectively [1]. The most important viral diseases of faba beans in the continent are necrotic yellows and stunt, caused mainly by two closely related nanoviruses, faba bean necrotic yellows virus (FBNYV) and faba bean necrotic stunt virus (FBNSV) (genus *Nanovirus*, family *Nanoviridae*). FBNSV was formerly a strain of FBNYV and was later reclassified as a distinct species due to its distinct serological, biological, and genomic properties [52]. FBNYV has a wider geographic distribution in West Asia, the Middle East, North and East Africa, and Europe (Spain), whereas FBNSV has been reported only from Ethiopia, Morocco, Azerbaijan, and Iran [14,53,54]. The two viruses cause similar symptoms on faba bean, which include severe plant stunting, yellowed, thick, and brittle leaves that show interveinal chlorotic blotches starting from the leaf margins, which becomes necrotic; and plant death within 5–7 weeks (Figure 1F). FBNYV and FBNSV symptoms in faba bean are more severe and damaging than those symptoms caused by other phloem-limited viruses like poleroviruses, luteoviruses, or mastreviruses [53]. During the 1991/1992 growing season, a severe epidemic of FBNYV led to 70–90% losses in faba bean production in Middle Egypt, the main faba bean-producing area in the country at that time [55]. Such severe epidemics were repeated in Egypt in the 1992/1993 and 1997/1998 growing seasons, with varying levels of severity [11]. Similarly, a high incidence of FBNSV (then considered a strain of FBNYV) which led to a near-total crop failure was reported in South Wello in northeast Ethiopia and the Ankober area northern Shewa [54,56]. Both FBNYV and FBNSV are transmitted efficiently by the aphids *A. pisum* and *A. craccivora*, and less efficiently by *Aphis fabae*, in a circulative persistent manner but not by seed. FBNYV also naturally infects other legume crops like cowpea in Syria and grass pea in Ethiopia but is economically important mainly on faba bean [14].

Several thousands of faba bean genotypes were screened for resistance in Ethiopia, Egypt, and Syria under field conditions, including artificial inoculation by viruliferous aphids, but no reliable source of resistance to FBNYV or FBNSV has been obtained [4,56,57]. Disease management therefore currently relies on other control measures. Cultural practices, such as delayed planting, rogueing, weed management, and chemical control of aphid vectors have been recommended to manage FBNYV [40]. Seed dressing with imidacloprid at 2.8 g a.i./kg of seeds reduced yield loss from 37% in untreated plots to 0% in treated ones [58]. The best FBNYV management successfully used in Egypt combines late planting in the growing season, a high seeding rate, one or two systemic insecticide sprays that are well-timed during the early stages of crop development, and early rogueing of infected plants in the growing season [4,59]. Since reliable natural resistance to FBNYV or FBNSV is not yet available in faba bean, novel approaches such as genetic engineering and genome editing should be explored for long-term disease control by host resistance. Challenges related to public acceptance of adopting such technologies should, in the meantime, be addressed by relevant national governments and other stakeholders.

## 6. Groundnut Rosette Disease

Groundnut, also called peanut, is a key crop grown throughout SSA, which contributes about 57% and 30% of global cultivation area and production, respectively, [1]. Top producing countries in SSA are Nigeria, Sudan, Senegal, Ghana, Chad, DRC, Tanzania, Guinea, Mali, and Burkina Faso. Groundnut rosette disease (GRD) is the most destructive disease of the crop in SSA, including several countries in East, West, Central, and Southern Africa. Reported in 1907 in Tanganyika (present-day Tanzania), GRD is the first legume virus disease reported in Africa [60]. GRD has not been reported outside of Africa. It is a disease complex caused by a tripartite synergistic interaction of groundnut rosette assistor virus (GRAV, genus *Polerovirus*, family *Solemoviridae*), groundnut rosette virus (GRV, genus *Umbravirus*, family *Tombusviridae*), and its satellite RNA (GRV-SatRNA) [15]. In this interaction, GRV and satellite RNA are encapsidated with the GRAV coat protein and also use it for transmission by the aphid vector, as they do not encode their own. The satellite RNA is assisted by GRV in replication. The satellite RNA is primarily responsible for disease symptoms [61].

The main GRD symptoms are stunting, shortened internodes, and reduced leaf size, resulting in a bushy appearance of the plants (Figure 1G,H). Two distinct symptom types, named ‘chlorotic rosette’ (chlorotic yellow leaf mosaic and rosette) and ‘green rosette’ (green leaf mosaic and rosette), are reported in different parts of Africa [15]. Yield losses are greatest in young plants and can reach 100%, resulting in complete crop failure when widespread infection occurs before flowering. Numerous epidemics of GRD have been reported in Africa, resulting in substantial crop losses [15,62]. The dramatic 1975 epidemic of groundnut rosette in northern Nigeria occurred on over 1 million ha of groundnut and destroyed an estimated 0.7 million ha, worth over USD 250 million [63]. The causal virus complex is readily transmitted by *Aphis craccivora* in a persistent manner but not by seed. Primary infection is introduced into the crop by viruliferous aphids likely derived from off-season groundnut volunteers and self-sown plants, whereas secondary spread occurs from sources within the crop by aphids [15].

Several approaches have been used to manage GRD. The use of insecticides to control aphid vectors has been effective [64], but most resource-poor small holder farmers in SSA cannot afford to use this option. Cultural practices such as early sowing in the season to take advantage of low aphid populations and maintaining good plant density without any gaps (aphids prefer widely spaced plantings for landing) have been shown to reduce rosette disease incidence [16,65]. Many farmers in SSA, however, do not adopt such recommendations; hence, limited success is achieved [15]. Rosette-resistant groundnut varieties provide the most economical and practical solution to control GRD in the field, and substantial efforts have been made to identify durable resistance from both early- and late-maturing groundnut varieties [16]. These resistance sources were used in breeding program throughout SSA, resulting in the development of several resistant cultivars released under various names in several countries, including Nigeria, Uganda, Malawi, Mozambique, Zambia, and Zimbabwe [16,66]. Future research should include epidemiological studies that help prevent future epidemics, including aphid population dynamics, survival and dispersal patterns, alternate hosts of the causal agents and/or the aphid vector, and steps to ensure the availability of seeds of resistant varieties [16].

## 7. Soybean Mosaic Diseases

Soybean (*Glycine max*) is an important legume cultivated worldwide as a source of edible oil and protein. In Africa, it is cultivated annually on about five million hectares with production of over seven million tons [1]. South Africa is the largest soybean producer in Africa, followed by Nigeria, while countries like Burkina Faso, Zambia, Uganda, Zimbabwe, Malawi, Ghana, Sudan, and Ethiopia are also important producers. Soybean mosaic virus (SMV, genus *Potyvirus*, family *Potyviridae*) is the most prevalent virus in many soybean-producing areas worldwide. Yield losses reported due to the virus range from 8 to 35% under natural conditions, but losses as high as 94% were reported in susceptible cultivars [67]. Disease symptoms include mosaic and mottling, leaf distortion, severe reduction in leaf size, and stunting. Many infected soybean cultivars often produce mottled or discolored seeds, which results in reduced seed quality and poor market value (Figure 1I). SMV is reported as the most common virus on soybean in Nigeria, South Africa, and Ethiopia, but the disease was considered of minor economic importance except in a few cases [7,17,18,19]. However, considering the vast area of soybean cultivation on the continent, only limited information is available on the status of virus diseases on the crop, which is very likely due to a lack of adequate virus surveys.

Apart from SMV, mosaic diseases caused by two other viruses on soybean in Africa have attracted some research attention. The first was cowpea mild mottle virus (CPMMV; genus *Carlavirus*, family *Betaflexiviridae*), which emerged as an important soybean disease in West Africa, including Nigeria [20] and Benin [21]. CPMMV causes severe symptoms in soybeans compared to cowpea, including mosaic, bud blight, dwarfing leaf, and stem necrosis [7,68]. The virus is also found to be the most prevalent in Benin, with the highest incidence, warranting research on its control [21]. CPMMV has in recent years received worldwide attention, as it seriously affects soybean production in major growing countries including Brazil and several countries in Asia [68]. Secondly, mosaic disease caused by soybean blotchy mosaic virus (SbBMV, genus *Cytorhabdovirus*, family *Rhabdoviridae*) is reported as an economically significant viral pathogen of soybean in South Africa. The virus was first identified in the northern and eastern parts of South Africa [18,22]. Surveys conducted in the early 1990s reported disease incidences reaching 32%, leading to yield losses of up to 20% depending on the cultivar [22]. The virus is transmitted by the leaf hopper *Paragallta caboverdensis* but is not seed-transmitted [23].

Since virus diseases on soybeans have not caused serious damage in Africa, little research was performed on their epidemiology and management. However, virus movement via seeds and vectors, monoculture, agricultural intensification, and climate change could alter the current balance, making the crop vulnerable to virus diseases. For example, evaluation of over 30 soybean cultivars released or in the pipeline in Nigeria revealed high susceptibility to CPMMV [19], suggesting the potential damage that can be caused. Research on CPMMV control methods such as planting date adjustment and the use of resistant varieties were conducted in Benin [21]. Future research on soybean viruses should focus on surveys to determine the status and impact of the viruses on the crop and breeding for resistance predominant in specific regions. CPMMV may deserve special attention in some regions of West Africa because of its widespread occurrence and ability to cause serious yield reduction, as reported from countries like Brazil [68].

## 8. Conclusions and Future Perspectives

Virus diseases affect all the major food legumes grown in Africa, inflicting serious yield losses with huge economic impact. Considerable progress has been made in the past decade in understanding the characteristics of causal viruses, their ecology, and diagnostics, which assisted in the development of appropriate disease control measures. Viruses most prevalent in cowpea, common beans, and soybeans cause mosaic diseases and are mostly both seed- and aphid-transmitted. These viruses have worldwide distribution, although their economic importance varies from one region to another. Seed transmission often provides primary inoculum contributing to disease epidemiology and is also important for virus movement from one location to another. On the other hand, viruses involved in GRD, chickpea stunt, and faba bean necrotic yellows and stunt are spread by aphids such as *Aphis craccivora* in a persistent manner. Whereas GRD distribution and economic significance are limited only to Africa, viruses causing chickpea stunt and faba bean necrotic yellows and stunt have their major impact in Africa, although they are also significant in some countries, particularly in West and Central Asia. For the majority of the significant virus diseases of food legumes in Africa, recommendations on control measures are available, although in some cases customizing them to specific local conditions may be necessary. In most cases, success is achieved when optimal combination of various control measures is integrated, taking local context into consideration. The major challenge, however, remains to ensure the adoption of such recommendations at a sufficiently large scale by most farmers to have an impact over large areas. Several factors, including farmers’ awareness and affordability of the recommended control measures, will influence their successful implementation. Virus diseases of food legumes that are currently important in Africa can spread to other continents by different means, including movement of infected seeds and seedlings due to germplasm movement or seed trade, or vector species along with such materials, causing damage to crops in the new environment. To prevent such introduction, quarantine systems should be strengthened at national or regional levels. In addition, exchange of information among the concerned parties in different countries on the prevention and control measures for diseases having similar epidemiology, and adopting them by customizing them to local conditions, will assist in managing the diseases on a global scale.

Much also remains to be performed in strengthening food legume virus research on the continent. For example, virus surveys appear to have covered only a part of the vast land area in which food legumes are cultivated. Hence, reports of disease occurrence, incidence, and economic impact reflect only the extent of virus surveys performed and are therefore likely to underrepresent the overall picture. For example, no virus is reported from six of the major soybean-growing countries (Ghana, Malawi, Benin, Burkina Faso, Togo, and Uganda), which cumulatively contribute to over 1.5 million hectares of cultivated area [1], although it is expected that at least SMV, which is highly seed-transmitted, occurs in these countries. Similarly, there is only scant information on the incidence and impact of virus diseases of cowpea and groundnut in much of southern Africa, which are significant growers of these crops. Furthermore, in many of the countries where legume virus surveys were conducted, studies were performed several years ago, and there is a gap in generating new data on disease epidemiology and control of the important diseases, particularly during the last decade or so. It is possible that new viruses are introduced or those reported before could have increased in incidence and impact or evolved into new strains in the intervening years. There is therefore a need to strengthen virus research by national programs and international agricultural research centers mandated with food legumes in Africa, with focus on large-scale virus surveys to precisely identify viruses and establish their relative importance and impact using modern diagnostic tools, and to develop appropriate control measures. The commonly used diagnostic methods are serological tests (particularly the enzyme-linked immunosorbent assay and the tissue blot immunoassay), the polymerase chain reaction (PCR) or reverse transcription (RT)-PCR, and next-generation sequencing, which are crucial in developing targeted and effective management options [7,40,69]. For important virus diseases with no specific recommended control methods, or for those in which recommendations made were not applicable for various reasons, there is a need to generate new information, including sources of host resistance and epidemiological data that support management decisions. In addition, the use of advanced crop improvement tools such as transgenics, CRISPR/CAS, and RNAi technologies should be explored to enhance plant resistance against specific virus diseases where conventional breeding is not successful. Public concern about the safe and ethical use of such technologies should be addressed by relevant regulatory authorities of national governments on a case-by-case basis. Control efforts should be integrated with other crop management practices so that the full potential of food legumes is harnessed for the benefit of hundreds of millions of African farmers who depend on them for their livelihood.

## Figures and Tables

**Table 1:** The annual production and cultivated areas of major food legumes in Africa.

Crop	Production (Tons)	Cultivated Area (ha)
Cowpea	9,472,693	15,159,849
Groundnut	16,316,670	17,657,657
Common bean	8,322,888	10,549,841
Chickpea	659,955	367,312
Faba bean	1,601,924	750,483
Soybean	7,337,956	5,028,487
Total	43,712,086	49,518,629

Source: FAOSTAT, 2023 [1].

**Table 2:** Properties and geographical distribution of the viruses that cause economically important diseases in food legumes in Africa.

Causal Virus	Crop and Disease	Particle Shape and Size	Genome Type and Size	Distribution in Africa [References]
bean common mosaic virus	common bean, mosaic	filamentous, 860 nm	ssRNA, ~10 kb	Throughout [5,6]
bean common mosaic necrosis virus	common bean, mosaic or black root	filamentous, 820 nm	ssRNA, ~10 kb	Throughout [5,6]
cowpea aphid-borne mosaic virus	cowpea mosaic	filamentous, 750 nm	ssRNA, ~10 kb	Throughout [7,8,9]
bean common mosaic virus-BlCM	cowpea, mosaic	filamentous, 820 nm	ssRNA, ~10 kb	Burkina Faso, Nigeria, Togo, Ghana, Kenya, Tanzania, Zambia [7,8,9]
cucumber mosaic virus	cowpea, mild mosaic	isometric, 29 nm	tripartite	Throughout [7,8,9]
chickpea chlorotic stunt virus	chickpea stunt	isometric, 28 nm	ssRNA, ~5.9 kb	Northeast and North [10,11]
beet western yellows virus	chickpea, stunt	isometric, 26 nm	ssRNA, ~5.7 kb	Northeast and North [12,13]
faba bean necrotic yellows virus	faba bean, necrotic yellows and stunt	isometric, 18 nm	ssDNA, octapartite, ~8 kb	Northeast and North [14]
faba bean necrotic stunt virus	faba bean, necrotic yellows and stunt	isometric, 18 nm	ssDNA, octapartite, ~8 kb	Ethiopia, Morocco, Azerbaijan, Iran [14]
groundnut rosette virus	groundnut rosette complex	isometric, 25 nm	ssRNA,~ 4.3 kb	Throughout [15,16]
groundnut rosette assistor virus	groundnut rosette complex	isometric, 25 nm	ssRNA, ~6 kb	Throughout [15,16]
groundnut rosette virus-satRNA	groundnut rosette complex	isometric, 25 nm	ssRNA, ~0.9 kb	Throughout [15,16]
soybean mosaic virus	soybean, mosaic	filamentous, 750 nm	ssRNA, ~9.6 kb	South Africa, Nigeria, Ethiopia, Egypt [17,18,19,20]
cowpea mild mottle virus	soybean, mosaic, leaf and stem necrosis	filamentous, 650 nm	ssRNA, ~8.2 kb	Nigeria, Benin, Kenya, Tanzania, Uganda [20,21]
soybean blotch mosaic virus	soybean, blotch mosaic	bacilliform, 350 × 93 nm	dsDNA, ~8 kb	South Africa [18,22,23]

## Data Availability

No new data were created or analyzed in this study. Only data available to the public was used. Data sharing is not applicable to this article.
